# Potentially inappropriate prescribing among older adults with hypertension in China: prevalence and related comorbidities across different outpatient settings

**DOI:** 10.3389/fphar.2024.1439230

**Published:** 2024-08-15

**Authors:** Jiaqi Chen, Shuang Wang, Lvliang Lu, Yujie Yang, Kai Wang, Jing Zheng, Zhijiang Zhou, Pi Guo, Yunpeng Cai, Qingying Zhang

**Affiliations:** ^1^ Department of Preventive Medicine, Shantou University Medical College, Shantou, Guangdong, China; ^2^ Shenzhen Health Development Research and Data Management Center, Shenzhen, Guangdong, China; ^3^ Shenzhen Institutes of Advanced Technology, Chinese Academy of Sciences, Shenzhen, Guangdong, China

**Keywords:** potentially inappropriate prescribing, hypertension, Beers Criteria, comorbidity, healthcare settings

## Abstract

**Purpose:**

Potentially inappropriate prescribing (PIP) is commonly encountered in older adults; yet, there is limited information on the occurrence of PIP among older adults with hypertension. This study aims to determine and compare the prevalence of PIP and its association with comorbidities in older adult outpatients with hypertension across hospitals and community health centers (CHCs).

**Methods:**

This 3-year (2015–2017) repeated cross-sectional study used electronic medical records from Shenzhen, China, involving 62 hospitals and 678 primary medical institutions. PIP was defined using the 2019 Beers Criteria. Older adults (≥65 years) with hypertension and at least one outpatient prescription were included. Modified Poisson regression analysis was used to assess the association between chronic comorbidities, healthcare settings, and PIP.

**Results:**

The prevalence of PIP in old adult outpatients with hypertension in 2015, 2016, and 2017 was 46.32%, 46.98%, and 46.58% in hospitals, with a sample size of 38,411, 46,235, and 50,495, respectively, and 29.14%, 26.66%, and 29.84% in CHCs, with a sample size of 26,876, 29,434, and 34,775 respectively. The top four most popular PIP in hospitals and CHCs was proton-pump inhibitors (PPIs), diuretics, benzodiazepines, and non-cyclooxygenase-selective non-steroidal anti-inflammatory drugs (NSAIDs), respectively. PIP was most associated with chronic gastrointestinal disease (adjusted prevalence ratio = 1.54, 95% confidence interval [CI] = 1.50–1.59) and mental and behavioral disorders (adjusted prevalence ratio = 1.49, 95% CI = 1.46–1.53) in hospitals and with mental and behavioral disorders (adjusted prevalence ratio = 1.99; 95% CI = 1.95–2.03) and musculoskeletal system and connective tissue disorders (adjusted prevalence ratio = 1.33; 95% CI = 1.31–1.36) in CHCs. The prevalence of PIP was significantly higher in hospital settings than in CHCs (adjusted prevalence ratio = 1.65; 95% CI = 1.63–1.66).

**Conclusion:**

Among older adult outpatients with hypertension in Shenzhen, PIP was more prevalent in hospitals than in CHCs. The comorbidities most strongly associated with PIP were chronic gastrointestinal disease and mental and behavioral disorders in hospitals and mental and behavioral disorders in CHCs. Clinical pharmacy integration needs to be considered to reduce inappropriate prescribing in this vulnerable population.

## 1 Introduction

As the global demographic shift results in a growing proportion of older individuals, enhancing the quality and safety of prescribing has emerged as a public health priority in the care of older adults (Page et al., 2010). Potentially inappropriate prescribing (PIP) refers to the prescribing, or under-prescribing, of medications in older persons that may potentially cause significant harm (O’Connor et al., 2012). Several studies documented that PIP is associated with the risk of hospital admission, emergency department visits, adverse drug events, and mortality (Alyazeedi et al., 2019; Liew et al., 2019; Cardwell et al., 2020; Mekonnen et al., 2021).

A variety of screening tools have been developed to identify PIP. These tools are valuable not only for clinical applications but also for research purposes, aiding in the investigation of the prevalence, risk factors, and outcomes associated with suboptimal prescribing (Zhu et al., 2023). A highly validated resource, the Beers Criteria for older adults, was initially developed in 1991 and has been updated in 2012, 2015, and 2019 by the American Geriatric Society (American Geriatrics Society Beers Criteria Update Expert, 2019). This tool has seen widespread application across various countries and regions (Liew et al., 2020).

The prevalence of PIP among older adults varies significantly worldwide. The highest rates are in Africa (47.0%), followed by South America (46.9%), Asia (37.2%), Europe (35.0%), North America (29.0%), and Oceania (23.6%) (Tian et al., 2023). A recent meta-analysis estimated the pooled prevalence of PIP at 39% (95% confidence interval [CI] = 25%–54%) in older Chinese people, demonstrating a high prevalence of PIP among older patients in China (Tian et al., 2022).

In 2020, the population of individuals with hypertension in China was reported to be 245 million, a figure that continues to increase (China and Hu, 2023). Hypertension has emerged as a significant public health issue within the country, necessitating urgent management measures. Older adults, in particular, require focused intervention efforts, with hypertension prevalence rates estimated at 55.8% among those aged 65 years and older (Ni et al., 2021).

Hypertension in older adults is influenced by several underlying mechanisms, such as mechanical hemodynamic changes, arterial stiffness, neurohormonal and autonomic dysregulation, and the aging kidney. Consequently, hypertension is linked to an increased risk of ischemic and hemorrhagic strokes, vascular dementia, Alzheimer’s disease, coronary artery disease and related events, atrial fibrillation, chronic kidney disease, and retinal disease (Oliveros et al., 2019). In 2021, a longitudinal study conducted in Jiangsu, China, found that 58.1% of hypertensive community-dwelling residents had comorbidities (Xu et al., 2022). Hypertension was the second most frequently coexisting disease, present in 57.7% of cases (Wu et al., 2022). In summary, while managing hypertension, it is crucial to also prevent and address comorbidities.

The treatment of hypertension often requires multiple medications for blood pressure control, cardiovascular risk management, and the treatment of common comorbidities. Older adults with hypertension in Shanghai have an average of 4.83 diseases and take an average of 5.13 medications daily, and the rate of polypharmacy, defined as the concurrent use of at least five medications, is estimated at 50.5% (Wu et al., 2022). Consequently, due to the higher number of required medications, older adults with hypertension are theoretically more likely to be subject to PIP than older adults in general. Although the prevalence of PIP in hypertensive older adults was estimated at 46% and 54.23% in small samples of African Americans and Brazilians (Bazargan et al., 2018; Rego et al., 2020), respectively, the existing prevalence still needs to be confirmed with larger samples and higher-quality studies. Additionally, the corresponding prevalence in China remains unknown.

Since 2015, China has implemented a hierarchical medical system (Zhou et al., 2021). Primary medical institutions, mainly community health centers (CHCs) in Shenzhen, provide comprehensive medical treatment, prevention, rehabilitation, and healthcare to the community. Hospitals receive referrals from lower-level medical institutions and offer specialized medical services for treating critical and complicated diseases. Although the system mandates that patients receive their first diagnosis at a CHC, they are still free to choose any medical institution for diagnosis and treatment (Bai et al., 2019). Due to the differences in function and medical resources between the two healthcare settings (Tai et al., 2006), there may be variations in outpatient prescribing patterns (Hu et al., 2016; Poudel et al., 2017). However, few studies have compared PIP between hospital and primary care settings.

Shenzhen is one of the first pilot cities in China to establish CHCs and has adopted a “hospital-run and hospital-management” strategy to expand and enhance both the quantity and quality of primary care services. This system has generally been effective in improving accessibility and continuity of care (Shi et al., 2015). Consequently, Shenzhen serves as a representative city for exploring differences in PIP between hospitals and CHCs.

Given the high prevalence of hypertension, the severity of comorbidities, and the variations in healthcare settings in China, this study aimed to determine and compare the prevalence of PIP and the impact of comorbidities on the prevalence of PIP among older adult outpatients with hypertension in hospitals and CHCs in Shenzhen.

## 2 Methods

### 2.1 Data source and study population

A 3-year repeated cross-sectional study (2015, 2016, and 2017) was conducted using data obtained from electronic medical records (EMRs) collected from 62 hospitals and 678 primary medical institutions by the Shenzhen Health Development Research and Data Management Center. Healthcare professionals, supervised by the Shenzhen Municipal Health Commission, collected, entered, updated, and de-identified all records. Diagnosis and prescription records were coded according to the International Classification of Disease 10th Revision (ICD-10) and Anatomical Therapeutic Chemical (ATC) classifications, respectively. Each patient who visited the aforementioned medical institutions was anonymized and assigned a unique identifier. The EMR database adhered to the guidelines of the World Medical Association Declaration of Helsinki (32nd term), and a waiver-of-consent protocol was approved by the Shenzhen Institute of Advanced Technology Institutional Review Board (No. SIAT-IRB-151115-H0084).

People were eligible for participation if they had a diagnosis of hypertension, were ≥65 years old before 1 January of each study year, and had at least one outpatient prescription recorded in the EMR during the study period. Outpatients with missing birth date or sex information were excluded.

Sample size was determined using the pwr package in R, based on a 46% PIP prevalence in hospitals and 34% in CHCs (Al Khaja et al., 2018; Bazargan et al., 2018), with α = 0.05 and power = 0.90. This required 523 hospital patients and 349 CHC patients, assuming a 1.5:1 ratio of hospital to CHC patients (Zhao et al., 2022).

To ensure stability and precision of the overall prevalence of PIP, the medical institutions involved in the study were required to be general hospitals and CHCs with at least one valid outpatient prescription record per month during the 3 years.

### 2.2 PIP definition

We used the 2019 version of the Beers Criteria (American Geriatrics Society Beers Criteria Update Expert, 2019) for assessing PIP in older adults with hypertension. The criteria consist of five evaluation sections: 1) medications that are potentially inappropriate in most older adults; 2) medications that are potentially inappropriate in older adults with certain conditions; 3) medications that should be used with caution in older adults; 4) medications that may cause potentially clinically important drug–drug interactions in older adults; and 5) medications that should be avoided or have their dosage reduced with varying levels of kidney function in older adults. We used only the first three sections of the 2019 Beers Criteria to calculate the prevalence of PIP in this study because the exact time of medication use and the creatinine clearance rates of the participants were not available in the EMRs. In addition, certain medications that require specific indicators or measurements, such as nitrofurantoin (creatinine clearance <30 mL/min), reserpine (>0.1 mg/day), and doxepin (>6 mg/day), as well as some medications listed in the criteria but not introduced in China, were not included in this study due to the lack of relevant records in the EMRs. Eventually, we developed a medication list (Supplementary Table S1) based on the first three sections of the 2019 Beers Criteria, which contained 48 items for assessing PIP from EMRs. The number of PIP (Table 1) represented the total number of different items that participants had prescribed during each study year, classified as 0, 1, 2, 3, 4, and ≥5. The prevalence of PIP was calculated by dividing the number of participants with at least one item prescription record by the total number of participants each year.

**TABLE 1:** Baseline characteristics and number of PIP for older adult outpatients with hypertension in hospitals and CHCs by year.

	Number (%) of participants							
	Hospital			P*	CHC			*p* ^†^
	2015 (n = 38,411)	2016 (n = 46,235)	2017 (n = 50,495)		2015 (n = 26,876)	2016 (n = 29,434)	2017 (n = 34,775)	
Age category^‡^								
65–69 years	12,168 (31.68)	15,038 (32.53)	16,442 (32.56)	<0.001	8,283 (30.82)	9,345 (31.75)	11,034 (31.73)	<0.001
70–74 years	10,022 (26.09)	11,489 (24.85)	12,621 (24.99)		7,459 (27.75)	7,811 (26.54)	9,202 (26.46)	
75–79 years	8,575 (22.32)	10,034 (21.70)	10,446 (20.69)		5,930 (22.06)	6,325 (21.49)	7,311 (21.02)	
80–84 years	4,906 (12.77)	6,062 (13.11)	6,839 (13.54)		3,265 (12.15)	3,715 (12.62)	4,503 (12.95)	
≥85 years	2,740 (7.13)	3,612 (7.81)	4,147 (8.21)		1,939 (7.21)	2,238 (7.60)	2,725 (7.84)	
Female^‡^	19,989 (52.04)	24,231 (52.41)	26,797 (53.07)	0.007	13,597 (50.59)	14,919 (50.69)	17,595 (50.60)	0.97
Polypharmacy (≥5 drugs)^‡^	12,519 (32.59)	15,180 (32.83)	16,141 (31.97)	0.002	6,237 (23.21)	7,247 (24.62)	9,216 (26.50)	<0.001
Comorbidities								
Cardiovascular disease^‡^	21,659 (56.39)	27,028 (58.46)	30,762 (60.92)	<0.001	14,466 (53.82)	16,291 (55.35)	19,737 (56.76)	<0.001
Cerebrovascular disease^‡^	9,907 (25.79)	12,047 (26.06)	13,456 (26.65)	0.01	5,325 (19.81)	6,146 (20.88)	7,690 (22.11)	<0.001
Diabetes mellitus	13,888 (36.16)	17,148 (37.09)	19,141 (37.91)	<0.001	9,793 (36.44)	10,888 (36.99)	13,019 (37.44)	0.04
Hyperlipidemia^‡^	15,602 (40.62)	19,723 (42.66)	22,276 (44.11)	<0.001	11,103 (41.31)	12,329 (41.89)	14,608 (42.01)	0.16
Cancer^‡^	2,376 (6.18)	3,010 (6.51)	3,346 (6.63)	0.02	1,336 (4.97)	1,515 (5.15)	1,925 (5.54)	0.007
Chronic liver disease^‡^	1,983 (5.16)	2,489 (5.38)	2,728 (5.40)	0.19	1,172 (4.36)	1,251 (4.25)	1,518 (4.36)	0.74
Chronic respiratory disease^‡^	5,558 (14.47)	7,425 (16.06)	8,605 (17.04)	<0.001	3,798 (14.13)	4,439 (15.08)	5,432 (15.62)	<0.001
Chronic kidney disease^‡^	2,974 (7.74)	3,743 (8.10)	4,384 (8.68)	<0.001	1,548 (5.76)	1,849 (6.28)	2,395 (6.89)	<0.001
Chronic gastrointestinal disease^‡^	862 (2.24)	1,087 (2.35)	1,217 (2.41)	0.24	399 (1.48)	430 (1.46)	569 (1.64)	0.18
Disorders of the musculoskeletal system and connective tissue^‡^	8,990 (23.40)	12,250 (26.50)	14,006 (27.74)	<0.001	8,326 (30.98)	9,401 (31.94)	11,214 (32.25)	<0.001
Chronic nervous system diseases^‡^	1,672 (4.35)	2,167 (4.69)	2,440 (4.83)	0.002	1,004 (3.74)	1,191 (4.05)	1,476 (4.25)	0.005
Mental and behavioral disorders^‡^	8,192 (21.33)	10,467 (22.64)	11,894 (23.56)	<0.001	5,993 (22.30)	6,736 (22.89)	8,198 (23.57)	0.001
Number of comorbidities^‡^								
0	3,970 (10.34)	4,231 (9.15)	4,157 (8.23)	<0.001	3,417 (12.71)	3,395 (11.53)	3,748 (10.78)	<0.001
1	8,514 (22.16)	9,037 (19.55)	9,260 (18.34)		5,551 (20.65)	5,899 (20.04)	6,850 (19.70)	
2	9,128 (23.76)	11,053 (23.91)	11,962 (23.69)		6,133 (22.82)	6,795 (23.09)	7,867 (22.62)	
3	7,488 (19.49)	9,654 (20.88)	10,728 (21.25)		5,299 (19.72)	5,850 (19.87)	7,098 (20.41)	
4	4,930 (12.83)	6,495 (14.05)	7,534 (14.92)		3,565 (13.27)	4,109 (13.96)	4,913 (14.13)	
≥5	4,382 (11.41)	5,765 (12.47)	6,854 (13.57)		2,911 (10.83)	3,386 (11.50)	4,298 (12.36)	
Number of PIP^‡^								
0	20,620 (53.68)	24,513 (53.02)	26,974 (53.42)	0.60	19,044 (70.86)	21,586 (73.34)	24,399 (70.16)	<0.001
1	9,386 (24.44)	11,420 (24.70)	12,369 (24.50)		5,756 (21.42)	5,918 (20.11)	7,589 (21.82)	
2	4,172 (10.86)	5,016 (10.85)	5,491 (10.87)		1,519 (5.65)	1,496 (5.08)	2,064 (5.94)	
3	1,997 (5.20)	2,455 (5.31)	2,635 (5.22)		392 (1.46)	314 (1.07)	501 (1.44)	
4	1,100 (2.86)	1,340 (2.90)	1,438 (2.85)		105 (0.39)	83 (0.28)	153 (0.44)	
≥5	1,136 (2.96)	1,491 (3.22)	1,588 (3.14)		60 (0.22)	37 (0.13)	69 (0.20)	
Total^‡^	17,791 (46.32)	21,722 (46.98)	23,521 (46.58)	0.15	7,832 (29.14)	7,848 (26.66)	10,376 (29.84)	<0.001

PIP, potentially inappropriate prescribing; CHC, community health center.

*p-values for comparison in hospitals between the 3 years, based on the chi-squared test.

^†^ *p*-values for comparison in CHCs between the 3 years, based on the chi-squared test.

^‡^Significant differences between hospitals and CHCs during the 3 years (*p*-value<0.05).

### 2.3 Polypharmacy definition

As an important influencing factor of PIP (Nishtala et al., 2014; Zhang et al., 2017; Alhawassi et al., 2019; Tian et al., 2021a; Khatter et al., 2021), polypharmacy was included in the study as a baseline characteristic. According to previous studies, polypharmacy and excessive polypharmacy were defined as the use of ≥5 and ≥10 different medications chronically in the year preceding the study year, respectively (Masnoon et al., 2017; Hoel et al., 2021). All the medications chronically used that were present in EMR prescription records were classified by the first three levels of the ATC code, listed in Supplementary Table S2. Common chronic drugs were directly counted, and other drugs had to have corresponding prescription records present for at least 4 of 8 consecutive weeks. We excluded prescriptions of topical drugs, contrast agents, radiopharmaceuticals, surgical excipients, and nutraceuticals. The screening, determination, counting process, and final results of the polypharmacy were reviewed by a clinical pharmacist.

### 2.4 Comorbidity definition

Referring to the current status of chronic comorbidities among older Chinese people (Zhou et al., 2019; Pazan and Wehling, 2021; Wu et al., 2022) and combined grouping of the first two levels of ATC according to the anatomical main groups and therapeutic subgroups, we classified hypertensive chronic comorbidities into 12 categories: cardiovascular disease, cerebrovascular disease, diabetes mellitus, hyperlipidemia, cancer, chronic liver disease, chronic respiratory disease, chronic kidney disease, chronic gastrointestinal disease, disorders of the musculoskeletal system and connective tissue, chronic nervous system disease, and mental and behavioral disorders. All relevant ICD-10 codes are given in Supplementary Table S3. Participants were considered to have the chronic comorbidity described above if they had any record of a relevant diagnosis during 3 years before the study year.

### 2.5 Statistical analysis

All analyses were stratified by diverse sources of prescriptions (hospitals and CHCs). We performed a descriptive analysis in 2015, 2016, and 2017 by counting the number of participants and calculating their percentages. The number of PIP and baseline information, which included age, sex, polypharmacy, and chronic comorbidity conditions, are all presented as categories. The chi-squared test was used to determine differences between years and healthcare settings. Modified Poisson regression analysis was used to determine the association between comorbidities, healthcare settings, and PIP (Zou, 2004; Martinez et al., 2017; Tamhane et al., 2017; Mwebesa et al., 2022). The presence of PIP during the study year was used as the binary dependent variable, with no PIP as the reference category. After conducting a univariate analysis, we included the year of study, age, sex, polypharmacy, and comorbidities in the multivariate modified Poisson regression model. Both unadjusted prevalence ratios (PRs) and adjusted PRs (aPRs), along with their 95% CIs, were estimated.

Data extraction and statistical analysis were performed using PL/SQL Developer v11.0.5 (Allround Automations Co.) and R v4.1.2 (R Development Core Team), respectively. A variance inflation factor >10 indicated collinearity between variables in the multivariate regression analysis. Two-sided *p* < 0.05 was considered statistically significant.

### 2.6 Sensitivity analysis

To account for potential biases due to the non-random allocation of participants to comorbidity and non-comorbidity groups, we conducted a sensitivity analysis. Participants in these groups may have different propensities to seek medical attention, which could bias the estimates. To mitigate this, we used propensity score matching to determine whether the observed association between comorbidity and PIP could be attributed to unmeasured confounders (D’Agostino, 1998).

The first step involved modeling the conditional probability of each hypertensive comorbidity to estimate propensity scores. The explanatory variables included the number of comorbidities per patient and the number of diagnoses per patient, both of which are independent risk factors for PIP. Participants with each comorbidity were then randomly sampled and matched with participants without the comorbidity at a 1:1 ratio based on the propensity score using the Matching package in R. In the final step, the same multivariate modified Poisson regression was performed to analyze the matched data from hospitals and CHCs.

## 3 Results

### 3.1 Baseline characteristics

**FIGURE 1:**
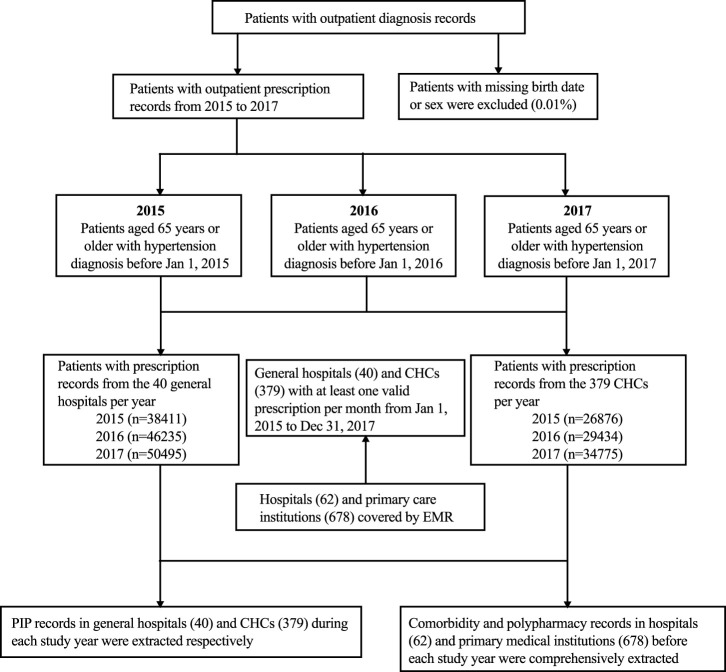
Flowchart for a 3-year repeated cross-sectional study. PIP, potentially inappropriate prescribing; CHC, community health center.

The number of older adult outpatients with hypertension and prescription records in 2015, 2016, and 2017 was 38,411, 46,235, and 50,495, respectively, in hospitals and 26,876, 29,434, and 34,775, respectively, in CHCs, significantly exceeding the minimum theoretical requirements calculated. The inclusion and exclusion of participants are shown in Figure 1. All baseline information is presented in Table 1. Except for sex and hyperlipidemia in CHCs and chronic liver disease and chronic gastrointestinal disease in both settings, the distribution of characteristics and comorbidities significantly differed among the 3 years for each setting. The overall distribution of age, sex, polypharmacy, and comorbidities other than diabetes mellitus differed between hospitals and CHCs.

The most prevalent chronic comorbidities in hospitals and CHCs were cardiovascular disease (56.39%–60.92% and 53.82%–56.76%, respectively), hyperlipidemia (40.62%–44.11% and 41.31%–42.01%, respectively), diabetes mellitus (36.16%–37.91% and 36.44%–37.44%, respectively), and cerebrovascular disease (25.79%–26.65% and 19.81%–22.11%, respectively). Nearly 90% of hospital-based and 88% of CHC-based participants had at least one chronic comorbidity.

### 3.2 Prevalence of PIP

Among older adult outpatients with hypertension who had prescription records in 2015, 2016, and 2017, the overall prevalence of PIP was 46.32%, 46.98%, and 46.58%, respectively, in hospitals and 29.14%, 26.66%, and 29.84%, respectively in CHCs (Table 1). In general, the prevalence of PIP was higher in hospitals than in CHCs (*p*-value < 0.001).

The items in the first section of the 2019 Beers Criteria, which identify medications that are potentially inappropriate in most older adults, and their prescribing prevalence are shown in the first part of Table 2. For 2015, 2016, and 2017, 37.16%, 37.78%, and 36.5% of participants were prescribed medications from this section in hospitals, and 23.91%, 21.14%, and 24.56% in CHCs, respectively. The most common PIP in 2015, 2016, and 2017 in hospitals was estazolam (5.90%, 5.96%, and 5.72%, respectively), diclofenac (6.08%, 5.95%, and 5.51%, respectively), omeprazole (3.39%, 5.42%, and 6.57%, respectively), pantoprazole (4.77%, 4.46%, and 4.46%, respectively), and rabeprazole (3.98%, 3.74%, and 3.93%, respectively). In CHCs, the most common PIP was estazolam (7.22%, 6.97%, and 7.93%, respectively), diclofenac (3.27%, 2.33%, and 3.5%, respectively), ibuprofen (3.16%, 2.65%, and 3.27%, respectively), omeprazole (2.69%, 1.88%, and 2.61%, respectively), and chlorpheniramine (2.30%, 1.77%, and 1.87%, respectively).

**TABLE 2:** Prevalence of PIP in older adult outpatients with hypertension in hospitals and CHCs by year.

PIP category	Drug class	Medication	Prevalence, n (%)					
			Hospital			CHC		
			2015	2016	2017	2015	2016	2017
Category I: medications that should be avoided by most older adults			14,272 (37.16)	17,466 (37.78)	18,429 (36.50)	6,427 (23.91)	6,221 (21.14)	8,539 (24.56)
Anticholinergics	First-generation antihistamines	Chlorpheniramine	572 (1.49)	640 (1.38)	521 (1.03)	618 (2.30)	520 (1.77)	651 (1.87)
		Cyproheptadine	109 (0.28)	109 (0.24)	84 (0.17)	86 (0.32)	86 (0.29)	98 (0.28)
		Diphenhydramine (oral)	10 (0.03)	16 (0.03)	19 (0.04)	5 (0.02)	8 (0.03)	10 (0.03)
		Promethazine	550 (1.43)	630 (1.36)	584 (1.16)	465 (1.73)	473 (1.61)	523 (1.50)
		Triprolidine	71 (0.18)	35 (0.08)	9 (0.02)	21 (0.08)	16 (0.05)	8 (0.02)
	Antiparkinsonian agents	Trihexyphenidyl	62 (0.16)	75 (0.16)	95 (0.19)	0 (0.00)	0 (0.00)	1 (0.00)
	Antispasmodics	Atropine (excludes ophthalmic)	112 (0.29)	108 (0.23)	124 (0.25)	8 (0.03)	13 (0.04)	12 (0.03)
		Belladonna alkaloids	163 (0.42)	167 (0.36)	65 (0.13)	79 (0.29)	65 (0.22)	69 (0.20)
		Hyoscyamine	483 (1.26)	521 (1.13)	510 (1.01)	245 (0.91)	171 (0.58)	220 (0.63)
		Scopolamine	5 (0.01)	8 (0.02)	6 (0.01)	3 (0.01)	2 (0.01)	11 (0.03)
Antithrombotics		Dipyridamole (oral short acting)	36 (0.09)	36 (0.08)	46 (0.09)	6 (0.02)	1 (0.003)	2 (0.01)
Cardiovascular	Peripheral alpha-1 blockers	Doxazosin	338 (0.88)	400 (0.87)	329 (0.65)	25 (0.09)	29 (0.10)	26 (0.07)
		Prazosin	69 (0.18)	76 (0.16)	77 (0.15)	0 (0.00)	0 (0.00)	0 (0.00)
		Terazosin	7 (0.02)	19 (0.04)	17 (0.03)	1 (0.004)	2 (0.01)	0 (0.00)
		Digoxin	529 (1.38)	636 (1.38)	691 (1.37)	19 (0.07)	11 (0.04)	23 (0.07)
		Nifedipine (immediate release)	355 (0.92)	479 (1.04)	652 (1.29)	152 (0.57)	134 (0.46)	191 (0.55)
		Amiodarone	355 (0.92)	431 (0.93)	490 (0.97)	25 (0.09)	19 (0.06)	30 (0.09)
Central nervous system	Antidepressants	Amitriptyline	52 (0.14)	69 (0.15)	77 (0.15)	1 (0.00)	0 (0.00)	1 (0.00)
		Clomipramine	2 (0.01)	2 (0.00)	2 (0.00)	0 (0.00)	0 (0.00)	0 (0.00)
		Paroxetine	125 (0.33)	123 (0.27)	166 (0.33)	5 (0.02)	3 (0.01)	4 (0.01)
	Antipsychotics	Perphenazine	21 (0.05)	22 (0.05)	22 (0.04)	0 (0.00)	0 (0.00)	0 (0.00)
		Chlorpromazine	6 (0.02)	8 (0.02)	17 (0.03)	2 (0.01)	0 (0.00)	1 (0.00)
		Penfluridol	0 (0.00)	1 (0.00)	0 (0.00)	0 (0.00)	0 (0.00)	0 (0.00)
		Sulpiride	10 (0.03)	12 (0.03)	17 (0.03)	0 (0.00)	0 (0.00)	0 (0.00)
		Tiapride	12 (0.03)	6 (0.01)	4 (0.01)	0 (0.00)	0 (0.00)	0 (0.00)
		Haloperidol	9 (0.02)	19 (0.04)	15 (0.03)	0 (0.00)	0 (0.00)	0 (0.00)
		Amisulpride	1 (0.003)	4 (0.01)	7 (0.01)	0 (0.00)	0 (0.00)	0 (0.00)
		Aripiprazole	14 (0.04)	14 (0.03)	15 (0.03)	0 (0.00)	0 (0.00)	0 (0.00)
		Clozapine	20 (0.05)	24 (0.05)	23 (0.05)	0 (0.00)	1 (0.00)	0 (0.00)
		Olanzapine	284 (0.74)	390 (0.84)	469 (0.93)	0 (0.00)	0 (0.00)	1 (0.00)
		Quetiapine	78 (0.20)	111 (0.24)	120 (0.24)	0 (0.00)	0 (0.00)	0 (0.00)
		Risperidone	51 (0.13)	61 (0.13)	61 (0.12)	0 (0.00)	0 (0.00)	0 (0.00)
		Ziprasidone	0 (0.00)	2 (0.00)	2 (0.00)	0 (0.00)	0 (0.00)	0 (0.00)
	Barbiturates	Phenobarbital	55 (0.14)	86 (0.19)	59 (0.12)	60 (0.22)	37 (0.13)	84 (0.24)
	Benzodiazepines	Alprazolam	1,332 (3.74)	1,798 (3.89)	2,188 (4.33)	19 (0.07)	43 (0.15)	86 (0.25)
		Estazolam	2,267 (5.90)	2,754 (5.96)	2,888 (5.72)	1,940 (7.22)	2,051 (6.97)	2,757 (7.93)
		Lorazepam	96 (0.25)	106 (0.23)	81 (0.16)	0 (0.00)	0 (0.00)	0 (0.00)
		Oxazepam	46 (0.12)	38 (0.08)	77 (0.15)	0 (0.00)	0 (0.00)	0 (0.00)
		Clonazepam	228 (0.59)	321 (0.69)	378 (0.75)	5 (0.02)	6 (0.02)	7 (0.02)
		Diazepam	508 (1.32)	580 (1.25)	647 (1.28)	240 (0.89)	247 (0.84)	316 (0.91)
	Nonbenzodiazepine and benzodiazepine receptor agonist hypnotics	Eszopiclone	26 (0.07)	100 (0.22)	168 (0.33)	0 (0.00)	4 (0.01)	4 (0.01)
		Zolpidem	8 (0.02)	14 (0.03)	17 (0.03)	0 (0.00)	1 (0.00)	8 (0.02)
		Zaleplon	460 (1.20)	565 (1.22)	647 (1.28)	22 (0.08)	27 (0.09)	52 (0.15)
Endocrine	Androgens	Testosterone	9 (0.02)	12 (0.03)	10 (0.02)	0 (0.00)	0 (0.00)	0 (0.00)
		Desiccated thyroid	2 (0.01)	1 (0.00)	0 (0.00)	0 (0.00)	0 (0.00)	1 (0.00)
		Estrogens with or without progestins	0 (0.00)	5 (0.01)	1 (0.00)	0 (0.00)	0 (0.00)	1 (0.00)
		Growth hormone	0 (0.00)	2 (0.00)	0 (0.00)	0 (0.00)	0 (0.00)	0 (0.00)
		Insulin (only short or rapid acting)	480 (1.25)	634 (1.37)	818 (1.62)	100 (0.37)	124 (0.42)	168 (0.48)
		Megestrol	12 (0.03)	19 (0.04)	17 (0.03)	0 (0.00)	0 (0.00)	0 (0.00)
	Sulfonylureas (long acting)	Glimepiride	699 (1.82)	785 (1.70)	822 (1.63)	265 (0.99)	317 (1.08)	431 (1.24)
		Glyburide	15 (0.04)	10 (0.02)	7 (0.01)	3 (0.01)	4 (0.01)	7 (0.02)
Gastrointestinal		Metoclopramide	353 (0.92)	392 (0.85)	402 (0.80)	95 (0.35)	79 (0.27)	100 (0.29)
	Proton-pump inhibitors (8 weeks^+^)	Omeprazole	1,302 (3.39)	2,504 (5.42)	3,316 (6.57)	724 (2.69)	552 (1.88)	907 (2.61)
		Rabeprazole	1,528 (3.98)	1,731 (3.74)	1,985 (3.93)	275 (1.02)	293 (1.00)	453 (1.30)
		Pantoprazole	1,834 (4.77)	2,143 (4.46)	2,254 (4.46)	224 (0.83)	282 (0.96)	506 (1.46)
		Lansoprazole	948 (2.47)	1,316 (2.85)	1,480 (2.93)	406 (1.51)	323 (1.10)	428 (1.23)
		Esomeprazole	921 (2.40)	1,247 (2.70)	1,587 (3.14)	128 (0.48)	155 (0.53)	219 (0.63)
		Lansoprazole	455 (1.18)	646 (1.40)	798 (1.58)	0 (0.00)	0 (0.00)	1 (0.003)
Pain medications		Meperidine	182 (0.47)	282 (0.61)	332 (0.66)	0 (0.00)	0 (0.00)	0 (0.00)
	Non-cyclooxygenase-selective NSAIDs (chronic use)	Diclofenac	2,335 (6.08)	2,751 (5.95)	2,784 (5.51)	880 (3.27)	685 (2.33)	1,217 (3.50)
		Diflunisal	28 (0.07)	30 (0.06)	30 (0.06)	0 (0.00)	0 (0.00)	0 (0.00)
		Ibuprofen	1,046 (2.72)	1,071 (2.32)	931 (1.84)	849 (3.16)	779 (2.65)	1,138 (3.27)
		Ketoprofen	64 (0.17)	195 (0.42)	201 (0.40)	0 (0.00)	0 (0.00)	1 (0.00)
		Meloxicam	143 (0.37)	130 (0.28)	103 (0.20)	10 (0.04)	11 (0.04)	11 (0.03)
		Oxaprozin	1 (0.00)	1 (0.00)	4 (0.01)	0 (0.00)	0 (0.00)	0 (0.00)
		Piroxicam	390 (1.02)	171 (0.37)	314 (0.62)	30 (0.11)	29 (0.10)	38 (0.11)
		Indomethacin	753 (1.96)	706 (1.53)	355 (0.70)	39 (0.15)	35 (0.12)	38 (0.11)
	Skeletal muscle relaxants	Chlorzoxazone	63 (0.16)	92 (0.20)	94 (0.19)	22 (0.08)	13 (0.04)	13 (0.04)
Genitourinary		Desmopressin	4 (0.01)	10 (0.02)	7 (0.01)	0 (0.00)	0 (0.00)	0 (0.00)
Category II: Medications that should be avoided by older adults due to drug–disease or drug–syndrome interactions			1,935 (5.04)	2,195 (4.75)	2,163 (4.28)	673 (2.50)	635 (2.16)	631 (1.81)
Cardiovascular	Heart failure	Cilostazol	3 (0.01)	3 (0.01)	4 (0.01)	0 (0.00)	0 (0.00)	0 (0.00)
		NSAIDs and COX-2 inhibitors	400 (1.04)	467 (1.01)	483 (0.96)	95 (0.35)	71 (0.24)	72 (0.21)
		Thiazolidinediones: pioglitazone and rosiglitazone	7 (0.02)	9 (0.02)	8 (0.02)	1 (0.00)	1 (0.00)	1 (0.00)
Central nervous system	Dementia or cognitive impairment	Anticholinergics	115 (0.30)	127 (0.27)	141 (0.28)	17 (0.06)	6 (0.02)	5 (0.01)
		Benzodiazepine	110 (0.29)	134 (0.29)	139 (0.28)	31 (0.12)	33 (0.11)	35 (0.10)
		Nonbenzodiazepine and benzodiazepine receptor agonist hypnotics: eszopiclone, zaleplon, and zolpidem	22 (0.06)	40 (0.09)	42 (0.08)	1 (0.00)	1 (0.00)	1 (0.00)
		Antipsychotics	90 (0.23)	118 (0.26)	142 (0.28)	1 (0.00)	0 (0.00)	1 (0.00)
	History of falls or fractures	Antiepileptics	64 (0.17)	82 (0.18)	83 (0.16)	19 (0.07)	10 (0.03)	13 (0.04)
		Antipsychotics	30 (0.08)	50 (0.11)	62 (0.12)	0 (0.00)	0 (0.00)	0 (0.00)
		Benzodiazepine	306 (0.80)	370 (0.80)	367 (0.73)	116 (0.43)	131 (0.45)	128 (0.37)
		Nonbenzodiazepine and benzodiazepine receptor agonist hypnotics: eszopiclone, zaleplon, and zolpidem	43 (0.11)	70 (0.15)	89 (0.18)	0 (0.00)	3 (0.01)	2 (0.01)
		TCAs	3 (0.01)	9 (0.02)	8 (0.02)	0 (0.00)	0 (0.00)	0 (0.00)
		SSRIs	52 (0.14)	71 (0.15)	72 (0.14)	1 (0.00)	0 (0.00)	0 (0.00)
		SNRIs	4 (0.01)	6 (0.01)	9 (0.02)	0 (0.00)	0 (0.00)	0 (0.00)
		Opioids	38 (0.10)	46 (0.10)	36 (0.07)	1 (0.00)	1 (0.00)	0 (0.00)
	Parkinson’s disease	Antiemetics: metoclopramide, prochlorperazine, and promethazine	18 (0.05)	17 (0.04)	12 (0.02)	3 (0.01)	1 (0.00)	2 (0.01)
		Antipsychotics	27 (0.07)	44 (0.10)	48 (0.10)	0 (0.00)	0 (0.00)	0 (0.00)
Gastrointestinal	History of gastric or duodenal ulcers	Non-COX-2-selective NSAIDs	828 (2.16)	885 (1.91)	836 (1.66)	350 (1.30)	347 (1.18)	343 (0.99)
Urinary tract	Urinary incontinence (all types) in women	Peripheral alpha-1 blockers: doxazosin, prazosin, and terazosin	1 (0.00)	0 (0.00)	1 (0.00)	0 (0.00)	0 (0.00)	0 (0.00)
	Lower urinary tract symptoms and benign prostatic hyperplasia	Strongly anticholinergic drugs	229 (0.60)	244 (0.53)	211 (0.42)	83 (0.31)	67 (0.23)	59 (0.17)
Category III: Medications to be used with caution in older adults			6,657 (17.33)	8,215 (17.77)	9,668 (19.15)	1,140 (4.24)	1,298 (4.41)	1,779 (5.12)
		Dabigatran	47 (0.12)	115 (0.25)	182 (0.36)	0 (0.00)	0 (0.00)	0 (0.00)
		Rivaroxaban	7 (0.02)	56 (0.12)	88 (0.17)	0 (0.00)	0 (0.00)	0 (0.00)
		Antipsychotics	461 (1.20)	602 (1.30)	704 (1.39)	2 (0.01)	1 (0.003)	2 (0.01)
		Carbamazepine	180 (0.47)	190 (0.41)	189 (0.37)	7 (0.03)	6 (0.02)	9 (0.03)
	Diuretics	Spironolactone	1,296 (3.37)	1,661 (3.59)	2,047 (4.05)	86 (0.32)	73 (0.25)	117 (0.34)
		Indapamide	1,212 (3.16)	1,541 (3.33)	1,756 (3.48)	240 (0.89)	234 (0.79)	322 (0.93)
		Torasemide	156 (0.41)	150 (0.32)	185 (0.37)	3 (0.01)	3 (0.01)	2 (0.01)
		Furosemide	1,051 (2.74)	1,505 (3.26)	1,885 (0.73)	16 (0.06)	17 (0.06)	38 (0.11)
		Hydrochlorothiazide	2,988 (7.78)	3,259 (7.05)	3,803 (7.53)	791 (2.94)	971 (3.30)	1,324 (3.81)
		Tolvaptan	0 (0.00)	3 (0.01)	2 (0.00)	0 (0.00)	0 (0.00)	0 (0.00)
		Mirtazapine	84 (0.22)	113 (0.24)	143 (0.28)	0 (0.00)	0 (0.00)	1 (0.00)
		Oxcarbazepine	70 (0.18)	137 (0.30)	133 (0.26)	0 (0.00)	0 (0.00)	0 (0.00)
	SNRIs	Duloxetine	57 (0.15)	60 (0.13)	81 (0.16)	0 (0.00)	0 (0.00)	0 (0.00)
	SSRIs	Citalopram	120 (0.31)	167 (0.36)	225 (0.45)	0 (0.00)	0 (0.00)	0 (0.00)
		Fluvoxamine	4 (0.01)	8 (0.02)	13 (0.03)	0 (0.00)	0 (0.00)	0 (0.00)
		Fluoxetine	230 (0.60)	287 (0.62)	239 (0.47)	1 (0.00)	1 (0.00)	3 (0.01)
		Paroxetine	125 (0.33)	123 (0.27)	166 (0.33)	5 (0.02)	3 (0.01)	4 (0.01)
		Sertraline	183 (0.48)	280 (0.61)	442 (0.88)	5 (0.02)	0 (0.00)	3 (0.01)
	TCAs	Amitriptyline	52 (0.14)	69 (0.15)	77 (0.15)	1 (0.00)	0 (0.00)	1 (0.003)
		Clomipramine	2 (0.01)	2 (0.00)	2 (0.00)	0 (0.00)	0 (0.00)	0 (0.00)
		Doxepin	22 (0.06)	49 (0.11)	58 (0.11)	0 (0.00)	0 (0.00)	1 (0.00)
		Tramadol	378 (0.98)	494 (1.07)	503 (1.00)	28 (0.10)	26 (0.09)	33 (0.09)
		Trimethoprim–sulfamethoxazole	10 (0.03)	20 (0.04)	9 (0.02)	10 (0.04)	5 (0.02)	2 (0.01)

PIP, potentially inappropriate prescribing; CHC, community health center; NSAIDs, non-steroidal anti-inflammatory drugs; COX-2, cyclooxygenase-2; SNRIs, serotonin–norepinephrine reuptake inhibitors; SSRIs, selective serotonin reuptake inhibitors; TCAs, tricyclic antidepressants.

For items in the second section of the Beers Criteria, medications that are potentially inappropriate in older adults with certain conditions (the second part of Table 2), the prescribing prevalence for 2015, 2016, and 2017 was 5.04%, 4.75%, and 4.28% in hospitals and 2.50%, 2.16%, and 1.81%, respectively, in CHCs. The most frequent PIP was non-cyclooxygenase-selective non-steroidal anti-inflammatory drugs (NSAIDs) prescribed for participants with a history of gastric or duodenal ulcers.

For items in the last section of the Beers Criteria, which include medications that should be used with caution in older people (the third part of Table 2), the prescribing prevalence for 2015, 2016, and 2017 was 17.33%, 17.77%, and 19.15% in hospitals and 4.24%, 4.41%, and 5.12%, respectively, in CHCs. Diuretics accounted for the largest proportion in both outpatient settings.

In summary, according to the first three sections of the 2019 Beers Criteria, the main medications prescribed in 2015, 2016, and 2017 in hospitals were proton-pump inhibitors (PPIs) (14.24%, 15.57%, and 15.64%, respectively), diuretics (13.44%, 13.46%, and 14.67%, respectively), benzodiazepines (9.74%, 10.28%, and 10.50%, respectively), and non-cyclooxygenase-selective NSAIDs (9.42%, 8.70%, and 7.91%, respectively). In CHCs, the main medications prescribed were benzodiazepines (7.58%, 7.30%, and 8.33%, respectively), non-cyclooxygenase-selective NSAIDs (6.25%, 4.93%, and 6.59%, respectively), PPIs (5.99%, 5.00%, and 6.48%, respectively), and diuretics (4.06%, 4.27%, and 4.95%, respectively).

### 3.3 Characteristics associated with PIP

Due to the presence of multicollinearity between the number of comorbidities and other covariables, we did not include it in the multivariate regression analysis.

In hospitals, the prevalence of PIP remained approximately the same in 2016 and 2017 compared to 2015 (2016: aPR = 1.02, 95% CI = 1.01–1.04; 2017: aPR = 1.03, 95% CI = 1.01–1.04) (Table 3). The prevalence of PIP was associated with age 75–79 years (aPR = 1.02; 95% CI = 1.01–1.03), 80–84 years (aPR = 1.08; 95% CI = 1.06–1.10), and ≥85 years (aPR = 1.14; 95% CI = 1.12–1.17) compared to 65–69 years, as well as female sex (aPR = 1.08; 95% CI = 1.07–1.09). As expected, polypharmacy was the strongest associated variable (aPR = 1.82; 95% CI = 1.80–1.85).

**TABLE 3:** Characteristics associated with PIP in hospitals and CHCs.

Covariate	Hospital		CHC	
	PR (95% CI)	aPR (95% CI)^‡^	PR (95% CI)	aPR (95% CI)^‡^
Year of study				
2015	1 (Ref.)	1 (Ref.)	1 (Ref.)	1 (Ref.)
2016	1.02 (1.00–1.03)	1.02 (1.01–1.04)*	0.91 (0.89–0.94)*	0.90 (0.88–0.93)*
2017	1.01 (0.99–1.02)	1.03 (1.01–1.04)^*^	1.02 (1.00–1.05)	1.02 (1.00–1.05)
Age group				
65–69 years	1 (Ref.)	1 (Ref.)	1 (Ref.)	1 (Ref.)
70–74 years	1.04 (1.03–1.06)*	0.99 (0.98–1.00)	1.04 (1.01–1.07)*	1.00 (0.97–1.03)
75–79 years	1.12 (1.10–1.15)*	1.02 (1.01–1.03)*	1.13 (1.09–1.17)*	1.06 (1.01–1.10)*
80–84 years	1.18 (1.16–1.20)*	1.08 (1.06–1.10)*	1.14 (1.10–1.18)*	1.09 (1.04–1.13)*
≥85 years	1.22 (1.20–1.23)*	1.14 (1.12–1.17)*	1.24 (1.16–1.27)*	1.24 (1.17–1.28)*
Female^†^	1.03 (1.02–1.03)*	1.08 (1.07–1.09)*	0.94 (0.91–0.97)*	0.96 (0.93–0.99)*
Polypharmacy (≥5 drugs)^†^	1.87 (1.84–1.90)*	1.82 (1.80–1.84)*	1.58 (1.55–1.62)*	1.38 (1.35–1.41)*
Comorbidities				
Cardiovascular disease^†^	1.24 (1.24–1.25)*	1.02 (1.00–1.01)*	1.21 (1.18–1.24)*	0.99 (0.97–1.01)
Cerebrovascular disease^†^	1.16 (1.15–1.17)*	1.02 (1.01–1.04)*	1.04 (1.00–1.08)*	0.96 (0.93–1.00)*
Diabetes mellitus^†^	1.13 (1.12–1.14)*	0.97 (0.95–0.98)*	1.12 (1.09–1.15)*	1.02 (1.00–1.05)*
Hyperlipidemia^†^	1.13 (1.12–1.14)*	1.02 (1.01–1.03)*	1.18 (1.15–1.20)*	0.98 (0.96–1.01)
Cancer^†^	1.13 (1.12–1.16)*	1.06 (1.03–1.08)*	1.09 (1.04–1.14)*	0.99 (0.93–1.04)
Chronic liver disease^†^	1.16 (1.14–1.18)*	1.05 (1.02–1.09)*	1.14 (1.08–1.19)*	0.99 (0.94–1.04)
Chronic respiratory disease^†^	1.23 (1.21–1.24)*	1.12 (1.11–1.14)*	1.19 (1.16–1.22)*	1.04 (1.01–1.07)*
Chronic kidney disease^†^	1.31 (1.29–1.34)*	1.16 (1.13–1.19)*	1.20 (1.13–1.26)*	0.99 (0.95–1.03)
Chronic gastrointestinal disease^†^	1.66 (1.63–1.71)*	1.54 (1.50–1.59)*	1.20 (1.12–1.30)*	1.01 (0.93–1.10)
Disorders of the musculoskeletal system and connective tissue^†^	1.20 (1.19–1.21)*	1.11 (1.09–1.12)*	1.41 (1.39–1.44)*	1.33 (1.31–1.36)*
Chronic nervous system diseases^†^	1.22 (1.19–1.25)*	1.02 (0.99–1.05)	1.04 (0.99–1.09)	0.93 (0.89–0.98)*
Mental and behavioral disorders^†^	1.62 (1.57–1.65)*	1.49 (1.46–1.53)*	2.08 (2.04–2.12)*	1.99 (1.95–2.03)*

PIP, potentially inappropriate prescribing; CHC, community health center; PR, prevalence ratio; CI, confidence interval; aPR, adjusted prevalence ratio.

*p < 0.05.

^†^Male, without polypharmacy, and without corresponding comorbidity were the reference category.

^‡^Adjusted for all other covariates.

In CHCs, the prevalence of PIP in older adult outpatients with hypertension reduced in 2016 compared to 2015 (aPR = 0.90; 95% CI = 0.88–0.93). The prevalence of PIP was also associated with age 75–79 years (aPR = 1.06; 95% CI = 1.01–1.10), 80–84 years (aPR = 1.09; 95% CI = 1.01–1.10), and ≥85 years (aPR = 1.24; 95% CI = 1.17–1.28) compared to 65–69 years and was reduced with female sex (aPR = 0.96; 95% CI = 0.93–0.99). Again, polypharmacy was associated with PIP (aPR = 1.58, 1.52–1.64).

### 3.4 Comorbidities associated with PIP

In hospitals, PIP was associated with all comorbidities examined except chronic nervous system diseases (Table 3). It was most associated with the comorbidities chronic gastrointestinal disease (aPR = 1.54; 95% CI = 1.50–1.59) and mental and behavioral disorders (aPR = 1.49; 95% CI = 1.46–1.53) in hospitals and mental and behavioral disorders (aPR = 1.99; 95% CI = 1.95–2.03) and disorders of the musculoskeletal system and connective tissue disease (aPR = 1.33; 95% CI = 1.31–1.36) in CHCs.

### 3.5 Comparative prevalence of PIP between hospitals and CHCs

The prevalence of PIP was higher in hospitals than in CHCs (aPR = 1.65; 95% CI = 1.63–1.66) (Table 4). Adjusted PRs for hospital compared to CHC prescribing ranged from 2.33 for chronic gastrointestinal disease to 1.39 for mental and behavioral disorders.

**TABLE 4:** Estimated PRs for PIP in hospitals compared to CHCs by comorbidity.

Comorbidity	PR (95% CI)	aPR (95% CI)
Participants with cardiovascular disease^†^	1.64 (1.62–1.66)*	1.67 (1.65–1.69)*
Participants with cerebrovascular disease^†^	1.76 (1.72–1.79)*	1.83 (1.80–1.87)*
Participants with diabetes mellitus^†^	1.64 (1.62–1.66)*	1.66 (1.63–1.68)*
Participants with hyperlipidemia^†^	1.59 (1.57–1.61)*	1.64 (1.62–1.67)*
Participants with cancer^†^	1.69 (1.63–1.75)*	1.78 (1.71–1.84)*
Participants with chronic liver disease^†^	1.66 (1.60–1.72)*	1.74 (1.67–1.81)*
Participants with chronic respiratory disease^†^	1.68 (1.65–1.71)*	1.72 (1.68–1.76)*
Participants with chronic kidney disease^†^	1.77 (1.72–1.82)*	1.84 (1.80–1.90)*
Participants with chronic gastrointestinal disease^†^	2.24 (2.17–2.31)*	2.33 (2.26–2.40)*
Participants with disorders of the musculoskeletal system and connective tissue^†^	1.48 (1.46–1.51)*	1.47 (1.45–1.49)*
Participants with chronic nervous system diseases^†^	1.90 (1.83–1.97)*	1.97 (1.89–2.05)*
Participants with mental and behavioral disorders^†^	1.39 (1.37–1.41)*	1.39 (1.37–1.41)*
All participants^†^	1.63 (1.61–1.65)*	1.65 (1.63–1.66)*

PIP, potentially inappropriate prescribing; CHC, community health center; PR, prevalence ratio; CI, confidence interval; aPR, adjusted prevalence ratio.

*p< 0.05.

^†^Adjusted for years, age, sex, polypharmacy, and other comorbidities.

### 3.6 Sensitivity analysis

Most of the estimated PRs for comorbidities were robust on propensity score matching (Supplementary Table S4) apart from diabetes mellitus (before matching: aPR = 0.97, *p* < 0.001; after matching: aPR = 0.97, *p* = 0.13) in hospitals and chronic respiratory disease (before matching: aPR = 1.04, *p* = 0.02; after matching: aPR = 1.02, *p* = 0.45) in CHCs. These discrepancies may be caused by randomness in sampling and matching.

## 4 Discussion

This 3-year repeated cross-sectional study used the 2019 Beers Criteria to examine PIP in older adults with hypertension from 2015 to 2017 across 40 hospitals and 379 CHCs in Shenzhen, China. The prevalence of PIP was significantly higher in hospitals than in CHCs. The top four most popular PIP in hospitals and CHCs was PPIs, diuretics, benzodiazepines, and non-cyclooxygenase-selective NSAIDs. In hospitals, PIP was associated with chronic gastrointestinal disease and mental and behavioral disorders, while in CHCs, it was associated with mental and behavioral disorders, as well as musculoskeletal and connective tissue disorders.

### 4.1 Comparison with previous studies

Compared with the prevalence of PIP in hospital settings (31%–34%) and CHCs (14%) in the general Chinese older population (also based on the 2019 Beers Criteria) (Fu et al., 2020; Tian et al., 2021b), we found a higher prevalence of PIP in hypertensive older people in Shenzhen, corresponding to more complex comorbidity profiles and medication patterns and indicating a serious situation of PIP in this vulnerable population.

Previous studies conducted in senior centers, resident supervision programs, and primary care settings reported the prevalence of PIP at 46%, 54%, and 34%, respectively, among older adults with hypertension (Al Khaja et al., 2018; Bazargan et al., 2018; Rego et al., 2020). The prevalence of PIP found in Shenzhen hospitals was similar to that in the first two settings, whereas the prevalence in Shenzhen CHCs was lower than that in the primary care setting study as the different PIP criteria Screening Tool of Older People’s Prescriptions (STOPP) was applied. In our study, the prevalence of PIP in hospitals was stable over the 3 years studied, whereas in CHCs, it fluctuated more significantly. As the quality of care enhances and the variety of medications increases in CHCs (Hu et al., 2016), the demographic structure of CHC patients is gradually evolving, and the pattern of drug prescribing needs to be further monitored.

The top four most frequent PIP overlapped in both medical settings: PPIs, diuretics, benzodiazepines, and non-cyclooxygenase-selective NSAIDs. PPIs (46%) and central nervous system active agents (18%) were the most commonly used potentially inappropriate medications in older African Americans with hypertension (Bazargan et al., 2018), which was similar to our study except for the higher prevalence of PPI prescription, as their study did not consider long-term use. A primary care setting study in Bahrain found that the skeletal muscle relaxant orphenadrine (9.05%), NSAIDs (7.08%), and PPIs (5.90%) were the most frequent PIP for hypertensive older people (Al Khaja et al., 2018), which shows a similar distribution of the main PIP in CHC settings in our study, except for orphenadrine, which had not been introduced in China.

### 4.2 Prevalent PIP

PPIs had the highest prevalence among PIP, with rabeprazole, omeprazole, and pantoprazole being the most commonly prescribed PPIs. These medications are primarily used to manage peptic ulcers and gastroesophageal reflux disease. Additionally, PPIs are often prescribed for cardiovascular and cerebrovascular patients as routine prophylactic when using aspirin and clopidogrel and in hematology, rheumatology, and immunology departments to mitigate the adverse effects of long-term, high-dose glucocorticoid therapy (Freedberg et al., 2017). However, according to the Beers Criteria, the long-term use of PPIs (beyond 8 weeks) should generally be avoided unless there are specific high-risk factors. Therefore, while PPIs can be clinically justified in certain cases, their use in hypertensive older adults should be approached with caution, and the duration of therapy should be carefully monitored to avoid unnecessary long-term exposure.

Diuretics were widely prescribed, the most common being hydrochlorothiazide. Diuretics are frequently combined with other antihypertensive medications, including angiotensin-converting enzyme inhibitors, angiotensin receptor blockers, and calcium channel blockers, to decrease fluid volume and prevent retention. This combination therapy is more effective at lowering blood pressure than monotherapy (Epstein, 2010). In addition to diuretics, PIP included doxazosin, prazosin, terazosin, clonidine, guanfacine, guanabenz, guanfacine, methyldopa, and reserpine (>0.1 mg/day), which could be used for blood pressure control.

The high prevalence of prescription of non-cyclooxygenase-selective NSAIDs and benzodiazepines can be explained by the high prevalence of chronic pain symptoms (Al-Ghamdi et al., 2021) and mental and behavioral disorders (Table 1) in the hypertensive older population.

When prescribing medications mentioned above in the target population, physicians should meticulously weigh the benefits against the risks and strengthen management practices.

### 4.3 Trends in PIP usage

We observed significant trends in PIP from 2015 to 2017 (Table 2). The use of PPIs (omeprazole and rabeprazole) and benzodiazepines (alprazolam and estazolam) increased in both hospitals and CHCs, highlighting the need to monitor long-term effects. Conversely, the use of non-selective NSAIDs (diclofenac and ibuprofen) decreased in hospitals, and the use of anticholinergic drugs (chlorpheniramine) decreased in both settings, reflecting the increased awareness of the side effects of these medications. The prescribing of antidepressants (citalopram and sertraline) and antipsychotics (olanzapine and quetiapine) increased in hospitals, corresponding to the growing burden of mental health issues (Bai et al., 2022). This underscores the necessity for more targeted mental health interventions and safer prescribing practices for elderly hypertensive patients. Additionally, the reduced use of NSAIDs and COX-2 inhibitors in patients with a history of ulcers indicates greater attention to patient medical histories.

### 4.4 Disparities in PIP prevalence between hospitals and CHCs

Most of the PIP was more common in hospitals than in CHCs, except for chlorpheniramine, promethazine, estazolam, and ibuprofen, which are commonly used for anti-allergy, anxiolytic, and anti-inflammatory purposes. PIP for chronic gastrointestinal disease, chronic nervous system disease, chronic kidney disease, and cerebrovascular disease was essentially not prescribed in CHCs as these are relatively severe conditions requiring treatment and prescription in hospitals. In the 3 years, China has been in the initial phase of establishing and perfecting the hierarchical medical system, and the majority of Chinese citizens were not accustomed to seeking initial diagnosis at primary medical institutions. Compared to the hospital setting, there was a notable imbalance in medical and pharmaceutical resources at CHCs. In both mild and severe cases, patients exhibit preference for diagnosing and treating directly at hospitals (Jin et al., 2015; Zhao et al., 2022). Most patients visit CHCs just to renew conventional long-term prescriptions or for minor ailment therapy, resulting in less exposure to PIP (Zeng et al., 2022). Therefore, the disparity in medical capabilities and the tendency of patients to seek a diagnosis were likely the main reasons for the variation in the prevalence of PIP between hospitals and CHCs.

### 4.5 Comorbidities and their association with PIP

According to results, 90% of older adults with hypertension have documented diagnoses of comorbid conditions. Hypertension has been previously associated with chronic pain, depression, and gastroesophageal reflux disease (Bazargan et al., 2018), which aligns with the comorbidities we identified as most closely associated with PIP. Not surprisingly, the PIP items included numerous commonly prescribed central nervous system drugs, gastrointestinal drugs, and analgesics (Supplementary Table S1), which correspond to a high prevalence of PIP in older hypertensive adults with these conditions.

While controlling blood pressure, the prevention and management of chronic comorbidities cannot be ignored. Clinicians may prescribe only in accordance with a “single-disease clinical guideline,” without considering the reality of multiple comorbidities and polypharmacy. This study provides evidence-based guidance for PIP, highlighting the need for individualized treatment plans considering comorbidities and medical settings.

### 4.6 Strategies for reducing PIP

A key strategy for reducing PIP includes using validated identification tools and collaborating with clinical pharmacists. Clinical pharmacists can conduct comprehensive medication reviews, identify PIP, and recommend safer alternatives. Urbańczyk et al. (2023) advocated for the wider adoption of clinical pharmacy practices in Central and Eastern Europe to optimize pharmacotherapy and improve patient outcomes. Their findings support integrating clinical pharmacists into healthcare teams to enhance medication management and safety, which could be beneficial in the Chinese context to reduce PIP prevalence among older adults with hypertension. Implementing these strategies requires supportive policies and training programs to equip healthcare providers with the necessary skills and resources. Future research should use the latest data and updated Beers Criteria, along with other widely accepted PIP criteria (e.g. STOPP), to ensure relevance and accuracy and explore the effectiveness of these strategies in diverse settings to reduce PIP and improve patient outcomes.

### 4.7 Strengths and limitations

The EMRs provided detailed access to the medical histories of participants for comorbidities. Our study comprehensively presents the situation of PIP in Shenzhen, featuring a large sample size, multiple temporal dimensions, and both hospital and CHC settings. We also included a detailed list of potentially inappropriate medications, covering three of the five categories in the 2019 Beers Criteria.

Additionally, the use of PRs is the most appropriate measure of association for analysis in cross-sectional studies. In particular, considering the high prevalence of PIP in our large sample, the use of odds ratio would overestimate the association in common outcomes, compromising the accuracy of the statistical analysis. Furthermore, the PR offers a more straightforward interpretation of the relative prevalence of PIP between different groups, making our findings more accessible to clinicians and policymakers.

However, the information obtained from systematic EMRs was limited, such as the lack of key diagnostic data on gait instability/falls and cognitive decline, the accurate duration of PIP, and the inability to identify the severity of disease and the prescribing preferences from different providers, which may have introduced potential biases in our results. Patients commonly select institutions based on their prescription needs, which could exacerbate the observed disparities between hospitals and CHCs. Additionally, the situation of each patient is complex, and determining whether a medication is appropriate or inappropriate from the list alone is difficult; it can only indicate the possibility of an inappropriate prescribing and provide a reference.

## 5 Conclusion

PIP was more common in hospitals than in CHCs among older adults with hypertension in Shenzhen. Chronic gastrointestinal disease and mental and behavioral disorders in hospitals, as well as mental and behavioral disorders and disorders of the musculoskeletal system and connective tissue in CHCs, were the top comorbidities most associated with PIP. Clinical pharmacy integration needs to be considered to reduce inappropriate prescribing in this vulnerable population.

## Data Availability

The original contributions presented in the study are included in the article/Supplementary Material; further inquiries can be directed to the corresponding authors.
