# Supplementary material for: Potentially inappropriate prescribing among older adults with hypertension in China: prevalence and related comorbidities across different outpatient settings
Source: Front Pharmacol. 2024 Aug 15;15:1439230. doi: 10.3389/fphar.2024.1439230 (PMC11357909; doi:10.3389/fphar.2024.1439230)
Supplement: Supplementary file 1 [file Table1.docx]

| **Supplementary Table 1 Classifications of PIP based on the 2019 Beers criteria** | | | | | |
| --- | --- | --- | --- | --- | --- |
| **Categories of PIP evaluation** |  | **PIP items** | | **Recommendation** | **Corresponding ATC code** |
| **Category I** | 1 | First-generation antihistamines:  Chlorpheniramine; Cyproheptadine; Diphenhydramine (oral); Promethazine; Triprolidine | | Avoid | R06AB04, R06AX02; D04AA32, R06AA02, D04AA10, R06AD52, R06AX07 |
|  | 2 | Antiparkinsonian agents: Trihexyphenidyl | | Avoid | N04AA01 |
|  | 3 | Antispasmodics: Atropine (excludes ophthalmic); Belladonna alkaloids; Hyoscyamine; Scopolamine | | Avoid | A03BA, S01FA01, A03CB03, A06AB30 |
|  | 4 | Antithrombotics: Dipyridamole, oral short acting (does not apply to the extended-release combination with aspirin) | | Avoid | B01AC07 |
|  | 5 | Peripheral alpha-1 blockers for treatment of hypertension: Doxazosin; Prazosin; Terazosin | | Avoid use as an antihypertensive | C02CA04, G04CA55, C02CA01, C02LE01, G04CA03 |
|  | 6 | Digoxin (for first-line treatment of atrial fibrillation or of heart failure) | | Avoid this rate control agent as firstline therapy for atrial fibrillation Avoid as first-line therapy for heart failure If used for atrial fibrillation or heart failure, avoid dosages >0.125 mg/day | C01AA05 |
|  | 7 | Nifedipine, immediate release | | Avoid | C08CA05 |
|  | 8 | Amiodarone | | Avoid as first-line therapy for atrial fibrillation unless patient has heart failure or substantial left ventricular hypertrophy | C01BD01 |
|  | 9 | Antidepressants, alone or in combination: Amitriptyline; Doxepin(>6 mg/day); Paroxetine | | Avoid | N06AA09, D04AX01, N06AA12, N06AB05 |
|  | 10 | Antipsychotics, first (conventional) and second (atypical) generation | | Avoid, except in schizophrenia or bipolar disorder, or for short-term use as antiemetic during chemotherapy | N05A |
|  | 11 | Barbiturates: Phenobarbital | | Avoid | N03AA02 |
|  | 12 | Benzodiazepines: (Short and intermediate acting:) Alprazolam; Estazolam; Lorazepam; Oxazepam (Long acting:) Clonazepam; Diazepam | | Avoid | N05BA12, N05CD04, N05BA06, N05BA56, N05BA04, N03AE01, N05BA01 |
|  | 13 | Nonbenzodiazepine, benzodiazepine receptor agonist hypnotics (ie, “Z-drugs”): Eszopiclone; Zaleplon; Zolpidem | | Avoid | N05CF04, N05CF03, N05CF02 |
|  | 14 | Androgens: Testosterone | | Avoid unless indicated for confirmed hypogonadism with clinical symptoms | G03BA03, G03EA02 |
|  | 15 | Desiccated thyroid | | Avoid | No corresponding ATC code, search by the drug name |
|  | 16 | Estrogens with or without progestins | | Avoid systemic estrogen (eg, oral and topical patch) | G03C, G03F |
|  | 17 | Growth hormone | | Avoid, except for patients rigorously diagnosed by evidence-based criteria with growth hormone deficiency due to an established etiology | H01AC |
|  | 18 | Insulin, sliding scale (insulin regimens containing only short- or rapid-acting insulin dosed according to current blood glucose levels without concurrent use of basal or long-acting insulin) | | Avoid | A10AB |
|  | 19 | Megestrol | | Avoid | G03AC05, G03DB02, L02AB01 |
|  | 20 | Sulfonylureas, long acting: Glimepiride; Glyburide (also known as glibenclamide) | | Avoid | A10BB12, A10BD04, A10BD06, A10BB01 |
|  | 21 | Metoclopramide | | Avoid, unless for gastroparesis with duration of use not to exceed 12 weeks except in rare cases | A03FA01 |
|  | 22 | Proton-pump inhibitors | | Avoid scheduled use for >8 weeks unless for high-risk patients (eg, oral corticosteroids or chronic NSAID use), erosive esophagitis, Barrett esophagitis, pathological hypersecretory condition, or demonstrated need for maintenance treatment (eg, because of failure of drug discontinuation trial or H2-receptor antagonists) | A02BC |
|  | 23 | Meperidine | | Avoid | N02AB02, N02AB52, N02AB72, N02AG03 |
|  | 24 | Non–cyclooxygenase-selective NSAIDs, oral: Diclofenac; Diflunisal; Ibuprofen; Ketoprofen; Meloxicam; Oxaprozin; Piroxicam | | Avoid chronic use, unless other alternatives are not effective and patient can take gastroprotective agent (proton-pump inhibitor or misoprostol) | M01AB05, M01AB55, N02BA11, M01AE01, R02AX02, M01AE51, M01AE03, M01AE53, M01AC06, M01AC56, M01AE12, M01AC01 |
|  | 25 | Indomethacin | | Avoid | C01EB03, M01AB01, M01AB51 |
|  | 26 | Skeletal muscle relaxants: Chlorzoxazone | | Avoid | M03BB03, M03BB53, M03BB73 |
|  | 27 | Desmopressin | | Avoid for treatment of nocturia or nocturnal polyuria | H01BA02 |
| **Category II** | 28 | Heart failure | Cilostazol | Avoid | B01AC23 |
|  | 29 |  | NSAIDs and COX-2 inhibitors | Use with caution in asymptomatic; avoid in symptomatic | M01A |
|  | 30 |  | Thiazolidinediones (pioglitazone, rosiglitazone) | Use with caution in asymptomatic; avoid in symptomatic | A10BG03, A10BG05, A10BG06, A10BG09, A10BG12, A10BD02, A10BD03, A10BD04 |
|  | 31 | Dementia or cognitive impairment | Anticholinergics | Avoid | A03E, A03AA, A03AB, R03AK, R03AL, R03BB |
|  | 32 |  | Benzodiazepines | Avoid | N03AE, N05BA, N05CD, N05CF |
|  | 33 |  | Nonbenzodiazepine, benzodiazepine receptor agonist hypnotics: Eszopiclone; Zaleplon; Zolpidem | Avoid | N05CF04, N05CF03, N05CF02 |
|  | 34 |  | Antipsychotics | Avoid | N05A |
|  | 35 | History of falls or fractures | Antiepileptics | Avoid except for seizure and mood disorders | N03A |
|  | 36 |  | Antipsychotics | Avoid unless safer alternatives are not available | N05A |
|  | 37 |  | Benzodiazepines | Avoid unless safer alternatives are not available | N03AE, N05BA, N05CD, N05CF |
|  | 38 |  | Nonbenzodiazepine, benzodiazepine receptor agonist hypnotics: Eszopiclone; Zaleplon; Zolpidem | Avoid unless safer alternatives are not available | N05CF04, N05CF03, N05CF02 |
|  | 39 |  | Antidepressants: TCAs; SSRIs; SNRIs | Avoid unless safer alternatives are not available | N06AA02, N06AA04, N06AA09,N06AA12, N06AB, N06CA03, N06AX16, N06AX21, N06AX17 |
|  | 40 |  | Opioids | avoid except for pain management in the setting of severe acute pain (eg, recent fractures or joint replacement) | N02A |
|  | 41 | Parkinson disease | Antiemetics: Metoclopramide; Prochlorperazine; Premethazine | Avoid | A03FA01, N05AB04, R06AD02 |
|  | 42 |  | Antipsychotics (except quetiapine, clozapine, pimavanserin) | Avoid | N05A(except N05AH04, N05AH02, N05AX17) |
|  | 43 | History of gastric or duodenal ulcers | Non-COX2-selective NSAIDs | Avoid unless other alternatives are not effective and patient can take gastroprotective agent | M01A(except M01AX17, M01AC06, M01AC56, M01AH01, M01AH02) |
|  | 44 | Urinary incontinence (all types) in women | Peripheral alpha-1 blockers: Doxazosin; Prazosin; Terazosin | Avoid in women | C02CA04, G04CA55, C02CA01, C02LE01, G04CA03 |
|  | 45 | Lower urinary tract symptoms, benign prostatic hyperplasia | Strongly anticholinergic drugs, except antimuscarinics for urinary incontinence | Avoid in men | A03E, A03AA, A03AB, R03AK, R03AL, R03BB |
| **Category III** | 46 | Dabigatran;  Rivaroxaban | | Use with caution for treatment of VTE or atrial fibrillation | B01AE07, B01AF01 |
|  | 47 | Antipsychotics; Carbamazepine; Diuretics; Mirtazapine; Oxcarbazepine; SNRIs; SSRIs; TCAs; Tramadol | | Use with caution | N05A, N03AF01, C03, N06AX11, N03AF02, N06AA02, N06AA04, N06AA09,N06AA12, N06AB, N06CA03, N06AX16, N06AX21, N06AX17, N02AX02 |
|  | 48 | Trimethoprim-sulfamethoxazole | | Use with caution in patients on ACEI or ARB and decreased creatinine clearance | J01EE01 |
| Abbreviations: PIP, potentially inappropriate prescribing; ATC, anatomical therapeutic chemical; NSAIDs, non-steroidal anti-inflammatory drugs; COX-2, cyclooxygenase-2; SNRIs, serotonin-norepinephrine reuptake inhibitors; SSRIs, selective serotonin reuptake inhibitors; TCAs, tricyclic antidepressants. | | | | | |

| **Supplementary Table 2 Chronic drug classification for polypharmacy** | |
| --- | --- |
| **ATC codes** | **Polypharmacy classifications** |
| A05A | Bile Therapy |
| A05B | Liver Therapy, Lipotropics |
| A10A | Insulins And Analogues |
| A10B | Blood Glucose Lowering Drugs, Excl. Insulins |
| A10X | Other Drugs Used In Diabetes |
| B01A | Antithrombotic Agents |
| C01A | Cardiac Glycosides |
| C01B | Antiarrhythmics, Class I And III |
| C01D | Vasodilators Used In Cardiac Diseases |
| C01E | Other Cardiac Preparations |
| C02A | Antiadrenergic Agents, Centrally Acting |
| C02C | Antiadrenergic Agents, Peripherally Acting |
| C03A | Low-Ceiling Diuretics, Thiazides |
| C03B | Low-Ceiling Diuretics, Excl. Thiazides |
| C03C | High-Ceiling Diuretics |
| C03D | Aldosterone Antagonists And Other Potassium-Sparing Agents |
| C04A | Peripheral Vasodilators |
| C05B | Antivaricose Therapy |
| C05C | Capillary Stabilizing Agents |
| C07A | Beta Blocking Agents |
| C08C | Selective Calcium Channel Blockers With Mainly Vascular Effects |
| C08D | Selective Calcium Channel Blockers With Direct Cardiac Effects |
| C09A | Ace Inhibitors, Plain |
| C09B | Ace Inhibitors, Combinations |
| C09C | Angiotensin II Receptor Blockers (Arbs), Plain |
| C09D | Angiotensin II Receptor Blockers (Arbs), Combinations |
| C10A | Lipid Modifying Agents, Plain |
| C10B | Lipid Modifying Agents, Combinations |
| G03C | Estrogens |
| G04B | Urologicals |
| G04C | Drugs Used In Benign Prostatic Hypertrophy |
| H03A | Thyroid Preparations |
| H03B | Antithyroid Preparations |
| H05B | Anti-Parathyroid Agents |
| J04A | Drugs For Treatment Of Tuberculosis |
| J05A | Direct Acting Antivirals |
| L01A | Alkylating Agents |
| L01B | Antimetabolites |
| L01C | Plant Alkaloids And Other Natural Products |
| L01D | Cytotoxic Antibiotics And Related Substances |
| L01E | Protein Kinase Inhibitors |
| L01F | Monoclonal Antibodies And Antibody Drug Conjugates |
| L01X | Other Antineoplastic Agents |
| L02A | Hormones And Related Agents |
| L02B | Hormone Antagonists And Related Agents |
| L03A | Immunostimulants |
| M01A | Antiinflammatory And Antirheumatic Products, Non-Steroids |
| M04A | Antigout Preparations |
| M05B | Drugs Affecting Bone Structure And Mineralization |
| N03A | Antiepileptics |
| N04A | Anticholinergic Agents |
| N04B | Dopaminergic Agents |
| N05A | Antipsychotics |
| N05B | Anxiolytics |
| N05C | Hypnotics And Sedatives |
| N06A | Antidepressants |
| N06B | Psychostimulants, Agents Used For Adhd And Nootropics |
| N06C | Psycholeptics And Psychoanaleptics In Combination |
| N06D | Anti-Dementia Drugs |
| N07A | Parasympathomimetics |
| P01B | Antimalarials |
| R03A | Adrenergics, Inhalants |
| R03B | Other Drugs For Obstructive Airway Diseases, Inhalants |
| R03C | Adrenergics For Systemic Use |
| R03D | Other Systemic Drugs For Obstructive Airway Diseases |
| Abbreviations: ATC, anatomical therapeutic chemical. | |

| **Supplementary Table 3 ICD-10 disease codes for querying diagnostics in EMRs** | | |
| --- | --- | --- |
|  | **Diagnosed disease and symptom** | **Corresponding ICD-10 code** |
| Comorbidity of hypertension (Table 1) | Cardiovascular disease | I05.0-I09.9, I11, I13, I20.0-I25.9, I27, I30.0-I52.9 |
|  | Cerebrovascular disease | I60.0-I61.9, I63.0-I63.9, I64, I65.0-I66.9, I67 |
|  | Diabetes mellitus | E10.0-E14.9 |
|  | Hyperlipidemia | E78.2, E78.4, E78.5 |
|  | Cancer | C00.0-C13.9, C15.0-C25.9, C30.0-C34.9, C37.0-C38.8, C40.0-C41.9, C43.0-C45.9, C47.0-C54.9, C56.0-C57.8, C58, C60.0-C63.8, C64.0-C67.9, C68.0-C68.8, C69.0-C75.8, C81.0-C86.6, C88.0-C97.9, D00.0-D00.2, D01.0-D01.3, D02.0-D02.3, D03.0-D06.9, D07.0-D07.2, D07.4, D07.5, D09.0, D09.2-D09.8, D10.0-D10.7, D11.0-D12.9, D13.0-D13.7, D14.0-D14.32, D15.0-D25.9, D26.0, D26.1, D27.0-D27.9, D28.0- D28.7, D29.0-D29.8, D30.0-D30.8, D31.0-D36.7, D37.0-D37.5, D38.0-D38.5, D39.1-D39.8, D40.0-D40.8, D41.0-D41.8, D42.0-D43.9, D44.0-D44.8, D45.0-D47.9, D48.0-D48.7, D49.2-D49.4, D49.6, D49.8, K31.7, K62.0, K62.1, K63.5, N60.0-N60.9, N84.0-N84.8, N87.0-N87.9, Z03.1, Z08.0-Z09.9, Z12.0-Z12.9, Z80.0-Z80.9, Z85.0-Z85.9, Z86.0 |
|  | Chronic liver disease | B18.0-B18.8, K70.0-K70.9, K72.1, K73.0-K73.9, K74.0-K74.6, K76.0, K77 |
|  | Chronic respiratory disease | A15.3, J18.0-J18.9, J41.0, J41.1, J41.8, J44.0-J44.9, J45.9, J47.0-J47.9, |
|  | Chronic kidney disease | E10.2, E11.2, E12.2, E13.2, E14.2, I12.0-I13.9, N02.0-N08.8, N15.0, N18.0-N18.9, Z49.0-Z49.3, Z52.4, Z99.2 |
|  | Chronic gastrointestinal disease | K25.4-K25.7, K26.4-K26.7, K27.4-27.7, K28.4-K28.7, K29.3-K29.5 |
|  | Disorders of the musculoskeletal system and connective tissue | M13.0-M13.9, M15.0-M19.9; M05.0-M06.9, M08.0-M08.8 |
|  | Chronic nervous system diseases | G00.0-G99.9 |
|  | Mental and behavioral disorders | F00.0-F99.9 |
| Condition specified in the criteria (Table 2-category II) | Heart failure | I50.0-I50.9 |
|  | Dementia or cognitive impairment | F03, F06.7, G10.X5 |
|  | Falls or fractures | D48.9, G82.2, M80, M84, M96.6, S02, S12, S22, S32, S42, S52, S62, S72, S82, S92, T90.2, T91.1-T91.2, T92.1-T92.2, T93.1-T93.2, W00-W19 |
|  | Parkinson disease | G20.X, G21.1-G21.9 |
|  | Gastric or duodenal ulcers | K25.0-K27.9 |
|  | Urinary incontinence (all types) | R32.X, N39.0-N39.9 |
|  | Lower urinary tract symptoms, benign prostatic hyperplasia | N30.0-N30.9, N34.0-N34.3, N40.X01 |
| Abbreviations: EMR, electronic medical records; ICD-10, international classification of diseases, 10^th^ revision. | | |

| **Supplementary Table 4 Estimate PRs for comorbidities associated with PIP after propensity score matching** | | | | |
| --- | --- | --- | --- | --- |
| **Comorbidity^a^** | **Hospital** | | **CHC** | |
|  | **Matched sample size (2015, 2016, 2017)** | **aPR (95%CI)^b^** | **Matched sample size (2015, 2016, 2017)** | **aPR (95%CI)^b^** |
| Cardiovascular disease | 24732, 32408, 34660 | 1.04 (1.01-1.08)^*^ | 19988, 22034, 25772 | 0.95 (0.91-1.00) |
| Cerebrovascular disease | 19724, 23134, 26004 | 1.03 (1.01-1.05)^*^ | 10004, 11746, 15042 | 0.93 (0.89-0.96)^*^ |
| Diabetes mellitus | 24912, 31994, 33382 | 0.97 (0.93-1.01) | 18450, 21006, 23754 | 0.97 (0.93-1.01) |
| Hyperlipidemia | 26410, 33694, 36664 | 0.99 (0.97-1.01) | 19874, 21952, 22770 | 1.05 (0.99-1.10) |
| Cancer | 4753, 6010, 6690 | 1.04 (1.00-1.08)^*^ | 2672, 3030, 3850 | 1.05 (0.99-1.13) |
| Chronic liver disease | 3966, 4974, 5456 | 1.05 (1.01-1.09)^*^ | 2344, 2502, 3036 | 1.03 (0.96-1.11) |
| Chronic respiratory disease | 10988, 14776, 17154 | 1.11 (1.09-1.14)^*^ | 3798, 8860, 10738 | 1.02 (0.98-1.06) |
| Chronic kidney disease | 5948, 7486, 8764 | 1.11 (1.07-1.14)^*^ | 3096, 3696, 4790 | 1.01 (0.95-1.07) |
| Chronic gastrointestinal disease | 1724, 2174, 2434 | 1.46 (1.40-1.52)^*^ | 798, 860, 1138 | 0.91 (0.80-1.03) |
| Disorders of the musculoskeletal system and connective tissue | 16224, 24400, 27662 | 1.14 (1.11-1.18)^*^ | 16620, 18506, 21774 | 1.16 (1.11-1.21)^*^ |
| Chronic nervous system diseases | 3344, 4334, 4880 | 1.01 (0.97-1.06) | 2008, 2382, 2952 | 0.92 (0.84-0.99)^*^ |
| Mental and behavioral disorders | 16324, 20408, 23606 | 1.45 (1.43-1.47)^*^ | 11972, 13400, 16156 | 1.83 (1.78-1.88)^*^ |
| Abbreviations: PIP, potentially inappropriate prescribing; CHC, community health center; aPR: adjusted prevalence ratio; CI: confidence interval.  ^a^ Significant difference in the number of comorbidities and frequency of diagnoses between participants with and without all the comorbidities. without corresponding comorbidity was the reference category.  ^b^ Adjusted for years, age, sex, polypharmacy and other comorbidities.  * *P*<0.05 | | | | |
